# Retraction: Silencing SUMO2 promotes protection against degradation and apoptosis of nucleus pulposus cells through p53 signaling pathway in intervertebral disc degeneration

**DOI:** 10.1042/BSR-20171523_RET

**Published:** 2021-10-06

**Keywords:** Intervertebral disc degeneration, Nucleus pulposus cells, p53 signaling pathway, SUMO2 gene silencing

This Retraction follows an Expression of Concern relating to this article previously published by Portland Press.

This article is being retracted from *Bioscience Reports* at the request of the Editor-in-Chief and the Editorial Board following receipt of a notification from a reader, alerting the Editorial Board to concerns in Figure 8A, which in part overlap with those published in *Journal of Cellular Biochemistry* (10.1002/jcb.26330). In addition, the Editorial Office has identified regions of duplication in Electron microscope IDD panel of Figure 1.

The authors have been contacted in regards to the Retraction, and have not responded to the Journal's queries and the concerns raised. Given the extent of the issues raised, the Editorial Board stand by the decision to retract the article.

